# Education on palliative care for Parkinson patients: development of the “Best care for people with late-stage Parkinson’s disease” curriculum toolkit

**DOI:** 10.1186/s12909-021-02964-6

**Published:** 2021-10-25

**Authors:** Dimitrios Gatsios, Angelo Antonini, Giovanni Gentile, Spyridon Konitsiotis, Dimitrios Fotiadis, Irini Nixina, Pille Taba, Christiane Weck, Stefan Lorenzl, Katharina Maria Lex, Piret Paal

**Affiliations:** 1 Department of Neurology, Medical School, University of Ioannina, GR-45110 Ioannina, Greece; 2 Unit of Medical Technology and Intelligent Information Systems, Department of Materials Science and Engineering, University of Ioannina, Ioannina, Greece; 3 Parkinson and Movement Disorders Unit, Study Center for Neurodegeneration, Department of Neuroscience, University of Padua, Padua, Italy; 4 Department of Biomedical Research, Institute of Molecular Biology and Biotechnology, Foundation for Research and Technology-Hellas, Ioannina, Greece; 5 Department of Neurology and Neurosurgery, Institute of Clinical Medicine|, University of Tartu, Tartu, Estonia; 6 Clinic of Neurology, Tartu University Hospital, Tartu, Estonia; 7 University Hospital Agatharied, Hausham, Germany; 8 Palliative Care Research Hub, WHO Collaborating Centre at the Institute of Nursing Science and Practice, Paracelsus Medical University in Salzburg, Salzburg, Austria

**Keywords:** Curriculum toolkit development, Continuing education, Palliative care, Parkinson’s disease

## Abstract

**Background:**

Palliative care education among all stakeholders involved in the care of patients with late-stage Parkinson’s disease is not adequate. In fact, there are many unmet educational and training needs as confirmed with a targeted, narrative literature review.

**Methods:**

To address these needs we have developed the “Best Care for People with Late-Stage Parkinson’s Disease” curriculum toolkit. The toolkit is based on recommendations and guidelines for training clinicians and other healthcare professionals involved in palliative care, educational material developed in recent research efforts for patients and caregivers with PD and consensus meetings of leading experts in the field. The final version of the proposed toolkit was drafted after an evaluation by external experts with an online survey, the feedback of which was statistically analysed with the chi-square test of independence to assess experts’ views on the relevance and importance of the topics. A sentiment analysis was also done to complement statistics and assess the experts positive and negative sentiments for the curriculum topics based on their free text feedback.

**Results:**

The toolkit is compliant with Kern’s foundational framework for curriculum development, recently adapted to online learning. The statistical analysis of the online survey, aiming at toolkit evaluation from external experts (27 in total), confirms that all but one (nutrition in advanced Parkinson’s disease) topics included, as well as their objectives and content, are highly relevant and useful.

**Conclusions:**

In this paper, the methods for the development of the toolkit, its stepwise evolution, as well as the toolkit implementation as a Massive Open Online Course (MOOC), are presented. The “Best Care for People with Late-Stage Parkinson’ s disease” curriculum toolkit can provide high-quality and equitable education, delivered by an interdisciplinary team of educators. The toolkit can improve communication about palliative care in neurological conditions at international and multidisciplinary level. It can also offer continuing medical education for healthcare providers.

**Supplementary Information:**

The online version contains supplementary material available at 10.1186/s12909-021-02964-6.

## Background

The World Health Organization (WHO) has made a strong commitment towards developing palliative care structures as an important component of integrated treatment for young and old patients at any stage of illness [1]. This commitment includes also Parkinson’s disease (PD) with patients and their caregivers having considerable (and mounting) unmet physical, psychosocial and spiritual needs, and experiencing great problems with coordination and continuity of care [2, 3]. To ensure optimal responses to palliative care needs, educating healthcare professionals, patients and caregivers is of major importance.

The extension of life expectancy and ageing of populations globally predicts rise in the prevalence of neurological and other chronic disorders casing related disability. It has been demonstrated that patients with chronic neurologic disorders suffer from the burden of disease progression without the hope for a cure. Therefore, symptom management and palliative care approaches should be discussed from the beginning of the illness.

Accordingly, the PD_Pal project is working on a new model of palliative care and novel PD management Guidelines that can be easily implemented and integrated in modern healthcare systems. Within this context PD_Pal also addresses the identified gaps in stakeholders’ education by designing, implementing and evaluating a postgraduate course linking PD specific modules to palliative care. Herewith we present the development of the “Best Care for People with Late-Stage Parkinson’s Disease” curriculum toolkit which is addressed not only to all healthcare professionals caring for patients with PD but also to the patients and their caregivers who also have unmet educational needs and limited knowledge of palliative care and its potential benefits. We specifically present the methods for the development of the curriculum toolkit which, as a last step, included an evaluation from external experts. At the time of the manuscript submission the course implementation as a Massive Open Online Course (MOOC) was still ongoing with pilot testing and evaluation from learners being the main future activities.

## Methods

**Fig. 1:**
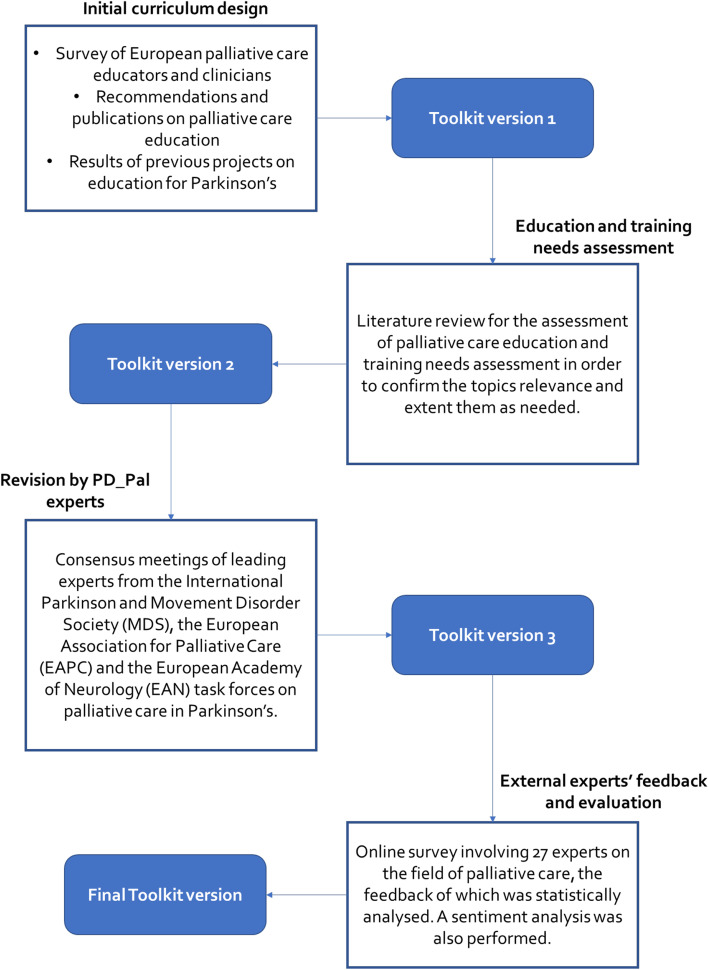
Curriculum toolkit development methodology

The development of the toolkit consisted of several steps (see Fig. 1). After identifying the lack of knowledge for palliative care and its interplay with advanced PD care as a problem to be addressed with a targeted educational intervention on international level, an initial needs’ assessment defined patients and their caregivers along with health care professionals as target audience. The first version of the toolkit was largely based on recent surveys, recommendations and guidelines for palliative care education and training of healthcare professionals, as well as on content recently developed for educating patients and caregivers on PD.

Then the needs of patients with PD, of their caregivers as well as of the healthcare professionals involved in the management of patients in the advanced stages of the disease were further analysed in respect to a narrative, yet targeted literature review in order to confirm the topics included in the curriculum and complete it as needed. The collection of the relative studies was not limited to a time range as palliative care education needs may be reported in older publications. We have utilized PubMed in which all relevant journals and conference proceedings are getting indexed. Within this context, a panel of three experts chose the most relevant quotes, which were used for querying. We chose quotes instead of keywords to ask a sufficiently focused research question. The selected quotes were ‘palliative care education Parkinson’s’ ‘Parkinson’s palliative care’ ‘palliative care education caregiver Parkinson’s’ ‘palliative care education patient Parkinson’s’ ‘palliative care education healthcare professionals Parkinson’s’ ‘advance care planning Parkinson’s’ and were used to produce the search terminology for this review, by considering all possible variations. Using these quotes, appropriate search queries were formulated, according to the specifications of PubMed. The research papers had to be written in English in order to be included in this review. The type of publication was not considered as a limitation, and all studies that were either published until October 2020 in international journals or conference proceedings were included.

After collecting the literature (in total 462 articles), and removing duplicates, the first and the last author screened the titles and the abstracts of all papers, aiming to apply a set of inclusion criteria which included reference to PD and palliative care or the advanced stage of the disease. Sixty-two articles qualified for full text assessment, 23 of theme were the most relevant. Two reviewers (the first and the last author) then went through the full text of the 23 manuscripts again and again to identify the educational gaps which informed the collection of patients’, caregivers’ and health care professionals’ needs.

After this narrative literature review, the second version of the curriculum toolkit was drafted. This second version was further assessed by Dr. Piret Paal, Prof. S. Lorenz, Prof. S. Konitsiotis, Prof. P. Taba, Prof. A. Antonini during both physical and virtual consensus meetings. The third version of the toolkit was consequently released, and it was evaluated with an online survey involving consenting, according to the General Data Protection Regulation (GDPR) provisions, experts on the field. Forty experts were invited by email to participate in the survey and the 27 that completed it (a 67,5% response rate) constituted the convenience sample.

Statistical, sentiment and analysis of the open-ended questions were employed for analysing the feedback collected with the online survey. The chi-square test of independence was used to analyse the frequency table (i.e. contingency table) formed by the distribution of frequencies of the survey responses for the importance, relevance and knowledge variables with respect to the different topics. The chi-square test evaluated whether there was a significant association between the categories of the responses and the topics. Residuals were calculated to assess each response value contribution to the topic, and then were turned into percentage contributions to the total chi-square score, for each cell. Pairwise z-test post hoc analysis with Bonferroni correction was performed to adjust standard residuals for multiple comparisons.

Finally, a sentiment analysis was conducted to display the sentiment scores of the word used in comments, with “affin” method [4], which uses an integer scale ranging from − 5 (very negative) to + 5 (very positive). Emotion classification is built on the NRC Word-Emotion Association Lexicon [5] which includes a list of English words and their associations with eight basic emotions (anger, fear, anticipation, trust, surprise, sadness, joy, and disgust) and two sentiments (negative and positive). Emotions and sentiments were then represented in % of occurrence within the text.

The statistical analysis was performed using R 4.0.3 (R Core Team, 2018). The package syuzhet (v1.0.4) was used for sentiment analysis and charts were made using the ggplot2 (v0.3.4). The full reproducible code is available in Supplementary Materials.

The analysis of the online survey findings led to the final curriculum toolkit.

## Results

### Initial curriculum design

The initial curriculum design was largely based on the results of a recent, relevant survey conducted among European palliative care educators and clinicians [6]. The evaluation of the resulting training content and course modules which demonstrated performance gain on all items considered [7] as well as the assessment of palliative care education related recommendations and publications [8–11] and the results of previous projects on education for PD [12, 13] led to the first version of the curriculum.

### Education and training needs assessment

This first version was revised according to the findings of the literature review which are presented herewith and are summarised in Table 1.

**Table 1:** Summary of educational and training needs identified with the narrative literature review

Stakeholders	Educational and training needs (attitude, skills, knowledge)	Literature
Patients and caregivers’	Learn how to navigate the PD journey	Jordan et al., 2020 [14]
	Being able to get personalized information	Fox et al., 2017 [15]
	Being able to choose the suitable palliative care setting	Sandsdalen et al., 2016 [16]
	Learn how to make decisions related to the place of death	Moens et al., 2015 [17]
Patients	Become knowledgeable of Advanced Care Planning (ACP)	Connor et al., 2015 [18]
	Comprehend what to expect and what PD management includes in the advanced stages	van der Eijk et al., 2012 [19]
Caregivers	Be trained to provide adequate care	McLaughlin et al., 2011 [20]
	Be informed about the availability and access to support groups and services	Olsson et al., 2016 [21]
	Learn how to cope with caregiver burden	Schrag et al., 2006 [22]
	Be taught how to handle the psychological impact	Hasson et al., 2010 [23]
	Be trained to manage emergencies	Goy et al., 2008 [24]
	Be informed in order to become a proxy for medical decisions	Sokol et al., 2019 [25]
	Get to understand the emotional, spiritual and bereavement domains	Aoun et al., 2010 [26]
	Comprehend death and be able to deal with it	Fox et al., 2017 [15]
	Be taught how to cope with pre-death grief	Carter et al., 2012 [27]
Healthcare providers	Be capable to efficiently communicate with patients and caregivers	Tuck et al., 2015, Walter et al., 2019 [28, 29]
	Be competent at information sharing	Miyasaki et al., 2016 [30]
	Be adept to provide primary palliative care	Lum et al., 2020, Robinson et al., 2017 [31, 32]
	Become highly skilled in pharmacotherapy	Katz et al., 2018 [33]
	Be able to guide shared decision-making around advance directives	Robinson et al., 2018 [34]
	Be knowledgeable of outpatient care models	Tarolli et al., 2019 [35]
	Comprehend the ethical challenges of ACP	Sokol et al., 2019 [25]

The shared needs of patients and their caregivers include the availability of simple, yet comprehensive tools for future planning. Essentially, they need practical guidance to navigate the PD journey [14]. They emphasize the personalization of the provided information and support [15]. They are also in need of comparative information on the advantages of palliative care quality when provided as inpatient care, within palliative units in nursing homes or at home [16] and which preferences should guide decisions for the place of death [17]. Patients indicated that improved education, shared decision making, and communication of Advanced Care Planning (ACP) [18] in order to be able to discuss advanced directives. Further education and emotional support, particularly regarding disease progression and their expectations, as well as advanced PD management [19] was also stressed as an important aspect.

The educational needs of caregivers include skills and support for coping with their difficulties when caring for their loved ones [20], increased knowledge of resources such as support groups programs and social services [21], learning how to cope with caregiver burden which rises significantly with disease progression [22]. They also need to improve their know-how for handling the impact of the disease including social isolation, loss of self-identity, feelings of helplessness, lack of control and physical deterioration of the patient’s and carer’s health [23]. Caregivers should also be better prepared to manage emergencies and learn how to anticipate physical challenges directly related to the debilitative course of the disease [24], be better prepared to speak as proxy for medical decisions [25] and comprehend the emotional, spiritual and bereavement domains [26], including how to deal with the death of the loved one [15] and how to manage pre-death grief [27].

The needs of healthcare providers caring for patients with PD, and eventually in need of palliative care, include training on communication (topics, timing, caregivers’ needs and management) [28, 29], education on proper information sharing [30], training on primary palliative care skills [31, 32] and mastering pharmacotherapy [33]. Healthcare professionals should also be able to build decision-making around advance directives and identify and manage symptoms of dying [34], as well as be trained on outpatient care models [35] and educated on the ethical challenges of ACP [25].

The second version of the toolkit that was informed by the literature review was further assessed during consensus meetings with experts that led to the definition of the third version which was assessed with an online survey from independent, external experts.

### Online survey – experts’ feedback and evaluation of the toolkit

A total of 27 experts participated to the survey. 89% of the sample was from Europe, 7.4% from Asia and 3.7% from North America. Neurologists accounted for 59.3% of the total sample, whilst Nurses for 11.1% and Palliative Care Physicians for 22.2%. 7.4% were Researchers. The age distribution of participants accounted for 62.9% of participants between 40 and 59, specifically 29.6% for age range 40–49, and 33.3% for age range 50–59. Only 3.7% of participants were among 20–29 and 3.7% above 70. Modal value of years of practice was 30–34 years (18.5%), however all the other intervals were similarily represented: 0–4 years, (14.8%); 5–9 years (7.4%); 10–14 years (14.8%); 15–19 years (14.8%); 20–24 years (7.4%); 25–29 years (14.8%); 30–34 years (18.5%); 35–39 years (3.7%); 40–44 years (3.7%).

No differences were found between the frequency distribution of the study participants’ roles and age (Χ^2^ = 23.7234, df = 15, *p* = .0699). No difference in proportion was found among the study participants’ roles and years of practice (Χ^2^ = 26.1562, df = 24, *p* = .3453).

**Fig. 2:**
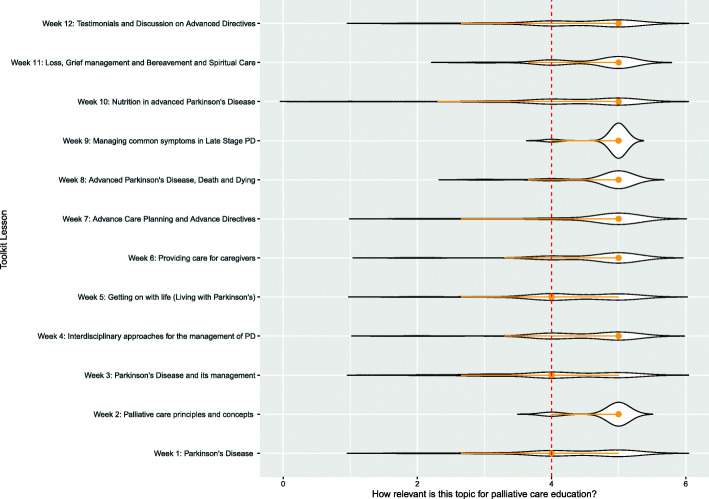
Relevance of topics constituting the Curriculum Toolkit

A chi-square test of independence was performed to examine the relation between the relevance score and the topics of the curriculum. The relation between these variables was significant, (χ^2^ = 67.0497, df = 44, *p* = .0141). Specifically, topic 10 “Nutrition in advanced PD” was more likely to be assessed as “not relevant” than the other topics. The most contributing cells to the Chi-square are “Not relevant/ Topic 10 - Nutrition in advanced PD” (15.04%), “Don’t mind/ Topic 3 – PD and its management” (10.262%), “Very relevant/ Topic 9 - Managing common symptoms in Late-Stage PD” (6%), “Very relevant/ Topic 5 - Getting on with life” (5.08%). These cells contribute about 31% to the total Chi-square score and thus account for most of the difference between expected and observed values (see Fig. 2). A post-hoc z-test on the adjusted residuals with Bonferroni correction revealed a significant difference only for “Nutrition in PD” as “not Relevant”, *p* < .05.

**Fig. 3:**
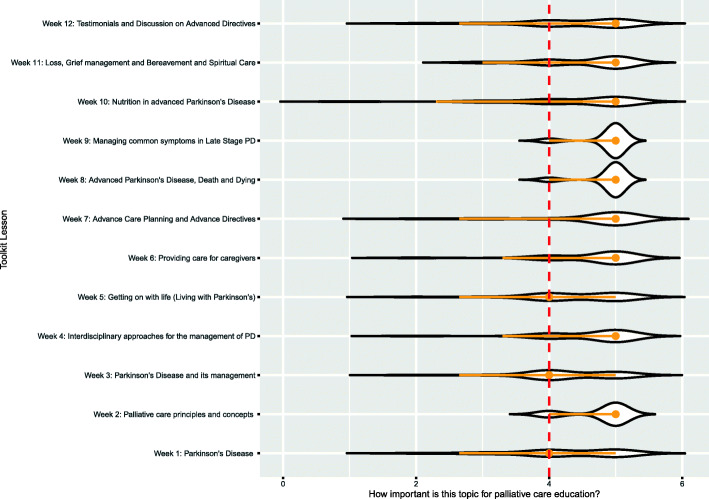
Importance of topics included in the Curriculum Toolkit

Importance results (Fig. 3), obtained with chi-square test of independence showed a significant relation between importance and topics’ variables, (χ^2^ = 65.2004, df = 44, *p* = .0205). Topic 10 - Nutrition in advanced PD, was more likely to be assessed as not important with residuals accounting for the 15.46% of the total χ^2^ score. Topic 3 – PD and its management was assessed as “very important” and “highly important”, with those cells contributing to the 15.24% of total chi square. The post-hoc z-test on the adjusted residuals with Bonferroni correction showed a significant difference only for “Nutrition in PD” as “not important”, *p* < .05.

**Fig. 4:**
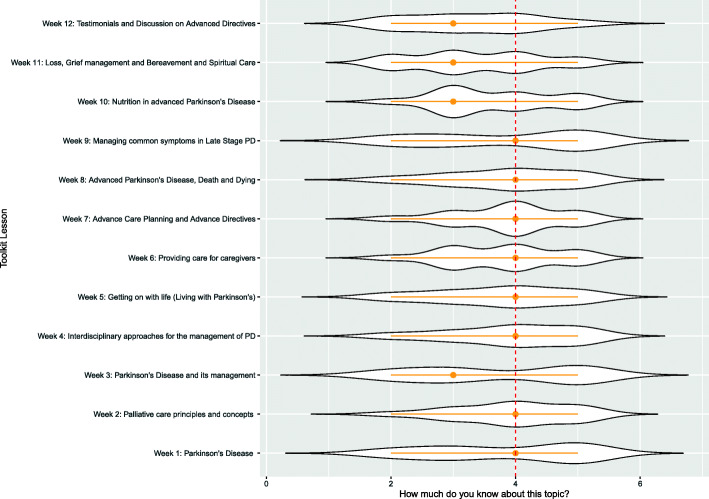
Experts’ self-assessment of their knowledge of the topics

Knowledge results (Fig. 4), obtained with chi-square test of independence showed a significant relation between the topic knowledge and the contents variables, (χ^2^ = 50.7118 df = 33 *p* = .0251). Topic 10 was more likely to be evaluated as “low-medium knowledge” with residuals accounting for the 7.82% of the total χ^2^ score. Topic 3 showed a lack of answer “medium-high” [4], varying of 11.75% from the total chi-square score. Topic 12 showed polarized answers, which shifted from the expected χ^2^ score by 4.43% for “low knowledge” values and a negative residuals variation of 5.88% for “very high knowledge” values. Topic 11, similarly, showed a gradual orientation of answers towards “low knowledge”, though with smaller effect, for which “low knowledge” accounted with a residual shift of 1.97%, and “very high knowledge” with a negative residuals’ shift of 3.69%. Topics 1, 7 and 9, were assessed as topics in which respondents considered themselves highly experienced, with “high” and “very high” answer much more represented than expected value (contribution to total X^2^ score ranging from 4.37 to 8.56%). The post-hoc z-test on the adjusted residuals with Bonferroni correction consistently highlighted a significant difference only for reduction of “medium-high knowledge” answer for Topic 3, *p* < .05.

According to the analysis of the experts’ feedback, that complemented the statistical findings, the selected topics are important and relevant. Some palliative care experts indicated that there is substantial overlap with geriatrics or palliative care curricula. Others, mainly neurologists, indicated that the PD_Pal curriculum could be expanded to discuss other neurological diseases as well.

In terms of including palliative care into healthcare professionals’ education, a physician from general hospital suggested: “*For healthcare professionals these topics must part of their education. For patients and informal caregivers appropriate timing is important - not too early, not too late.*” (ID_021) One expert pointed out that there might be some overlap with geriatrics or palliative care specialist training: “*General content is part of Geriatrics but specific features for PD patients are rather important, so these should be handled separately too*” (ID_023). Another expert suggested: “[Palliative care] *should be part of neurology education at every level*” (ID_015). In terms of improvements, experts suggested adding some different literature: “*The articles chosen may not be the most evidence-based or recent evidence.*” (ID_015). One expert pointed out the need for discussing diversity in healthcare services: “*There should also be some discussion about care delivery in under-resourced settings*” and possible country-level differences in Advanced Care Planning: “*Is there a possibility to include some info on ACPs in different regions?*” (ID_011). Some experts were concerned about the symptom control: “T*his is challenging and if education is directed to Palliative Care specialists, there are many meds and approaches that are not familiar to them.*” (ID_015). The topic of spirituality “*needs more time*.” (ID_013). Overall, positive feedback was given regarding including the patient and family experiences: “*Wonderful to include patient and family perspectives on this matter*” (ID_015).

**Fig. 5:**
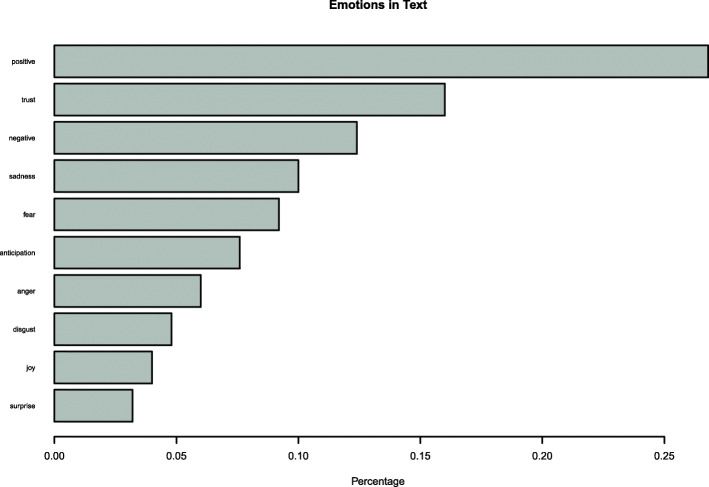
Sentiment analysis

A sentiment analysis was also conducted. The comments reported to the PD_Pal toolkit curriculum showed a significant percentage (χ^2^ = 109.54, df = 9, *p*-value < .001), of positive sentiment *n* = 63 (27.63%) and trust, *n* = 36 (15.79%). Negative sentiment and emotions accounted for a non-significant proportion (Fig. 5).

This extensive analysis led to the final curriculum toolkit, which is available in https://www.pdpal.eu/courses .

## Discussion

Our curriculum is compliant with Kern’s foundational framework for curriculum development [36] which was recently adapted to online learning [37]. Specifically, the identified lack of palliative care education among all stakeholders involved in the care of patients with late-stage PD is being addressed with an evidence -based curriculum toolkit. The initial assessment of educational needs is based on guidelines for palliative care education and previous curriculums for PD (step 1 in Kern’s six-step approach for curriculum development for medical education). These needs are further informed by a narrative literature review (step 2). Accordingly, the learning objectives and the content addressing learner’s attitude, knowledge and skills are defined and finalized after revisions and consensus meetings with leading experts on the field, as well as a targeted online survey aiming at curriculum evaluation (step 3).

In their majority the topics and their objectives and content were considered as highly relevant and useful. The major modification resulting from the toolkit evaluation was the integration of the previously dedicated topic on “Nutrition in advanced PD” in the “Managing common symptoms in Late-Stage PD” topic. This discrepancy can be attributed to the fact that in some countries tube feeding is not a common practice especially for patients with PD, even in the advanced stages. The objectives and content were not modified though as the available literature indicates that decisions regarding artificial nutrition and hydration are among the most common and complex decisions facing clinicians when patients with neurologic diseases have swallowing difficulties. Such decisions often involve incomplete clinical information, strong and often conflicting patient, caregiver, and healthcare professional knowledge and attitudes as well as diverse cultural and religious views that affect the final decision. Another adjustment that is made in the final toolkit according to the analysis of the experts’ feedback is that the topic “Spiritual Care” was assigned its own dedicated module while previously it was part of the broader “Grief management and Bereavement” topic.

The toolkit can be used in various ways. It can be the basis for traditional teaching through a series of workshops and seminars. It can also complement traditional teaching in order to enable participants delve into additional information for some of the topics. For instance, neurologists may only be interested in those topics related with palliative care. Educators can also choose which topics they want to include in their lessons and adjust accordingly. Within PD_Pal project the toolkit will be implemented as a Massive Open Online Course (MOOC) [38] which is a modern approach in medical education [39] increasingly gaining interest [40]. This is step 4 in Kern’s six-step approach for curriculum development for medical education. Specifically, the learner centric and focused on usability and scalability Open edX was adopted. It encourages active learning which is a modular approach to learning through interaction. And unlike traditional teaching, it supports self-paced learning. In fact, a weekly workload of 4 h, which is adopted also in the curriculum toolkit, at each learner’s pace, seems to facilitate course participation even for busy clinicians who tend to consider these open courses as relevant and potentially valuable means of post-graduate education [41]. Importantly, to address the variance in experience and expertise among healthcare professionals in palliative care and Parkinson’s disease topics, all learners can choose which topics are of their interest and be educated only on them: topics are designed to also be used as standalones.

The content for each topic which includes slides with transcripts, introductory and explanatory videos, additional resources, literature, and self-assessment exercises is being developed by a team of designated researchers working for PD_Pal project and is carefully revised by several experts. Then the course coordinating center which is the Paracelsus Medical University in Salzburg aligns all topics to ensure their consistency and smooth integration. This process involves several parties and is time-consuming due to various levels of revisions and quality checks.

Consistent with findings from [42], we expect as positive outcomes of the course the efficient set-up and content of the course, the pedagogical approach and the consistent international focus. The major benefits of the MOOC presenting the toolkit will include mutual learning and exchange of palliative care experiences and know-how from around the world that would have been impossible to achieve in traditional learning contexts. We also anticipate the lack of more practical case studies. Advanced, in person, follow-up courses on certain topics are needed for fully achieving the acquisition of skills. In fact, our toolkit and its implementation as different topics allow palliative care educators to use either some parts of it or as a whole.

The curriculum will be piloted in order to be revised according to learners’ experience and feedback, prior to full implementation (step 5 in Kern’s approach). Evaluation will be an ongoing process and after each course deployment revisions will be applied as needed. The MOOC approach calls for additional assessment of usability, sustainability and satisfaction with the technology (step 6).

## Conclusions

The “Best Care for People with Late-Stage Parkinson’ s disease” curriculum toolkit was developed based on evidence and with a carefully designed methodology in order to provide high-quality and equitable education which will be delivered by an interdisciplinary team of educators. Implemented as a MOOC, it has the potential to educate patients and their families, informal and formal caregivers, medical and social profession students, healthcare providers, and eventually anyone interested in palliative care in PD. It can also be adapted to other neurological conditions. Overall, the toolkit has the potential to improve communication about palliative care at international level and at the same time improve health literacy for patients and their caregivers and offer continuing medical education for healthcare providers.

## Supplementary Information

**Additional file 1.**

## Data Availability

The datasets used and/or analysed during the current study are available from the corresponding author on reasonable request.

## References

[1] Organization WH (2014). Strengthening of palliative care as a component of integrated treatment throughout the life course. J Pain Palliative Care Pharmacotherapy.

[2] Miyasaki JM, Kluger B (2015). Palliative care for Parkinson’s disease: has the time come?. J Curr Neurol Neurosci Rep.

[3] Van Vliet LM, Gao W, DiFrancesco D, Crosby V, Wilcock A, Byrne A (2016). How integrated are neurology and palliative care services? Results of a multicentre mapping exercise. BMC Neurol.

[4] Nielsen FÅ (2011). A new ANEW: Evaluation of a word list for sentiment analysis in microblogs. Proceedings of the ESWC2011 Workshop on ‘Making Sense of Microposts’: Big things come in small packages 718 in CEUR Workshop Proceedings.

[5] Mohammad S (2018). Obtaining reliable human ratings of valence, arousal, and dominance for 20,000 english words. Proceedings of the 56th Annual Meeting of the Association for Computational Linguistics (Volume 1: Long Papers).

[6] Paal P, Brandstötter C, Lorenzl S, Larkin P, Elsner F (2019). Postgraduate palliative care education for all healthcare providers in Europe: results from an EAPC survey. J Palliative Support Care.

[7] Paal P, Brandstötter C, Bükki J, Elsner F, Ersteniuk A, Jentschke E, Stähli A, Slugotska I (2020). One-week multidisciplinary post-graduate palliative care training: an outcome-based program evaluation. BMC Med Educ.

[8] Best M, Leget C, Goodhead A, Paal P (2020). An EAPC white paper on multi-disciplinary education for spiritual care in palliative care. J BMC Palliative Care.

[9] Brighton LJ, Koffman J, Hawkins A, McDonald C, O'Brien S, Robinson V, Khan SA, George R, Higginson IJ, Selman LE (2017). A systematic review of end-of-life care communication skills training for generalist palliative care providers: research quality and reporting guidance. J Pain Symptom Manag.

[10] Gamondi C, Larkin P-J, Payne S (2013). Core competencies in palliative care. Eur J Palliat Care.

[11] Mason SR, Ling J, Stanciulescu L, Payne C, Paal P, Albu S, Noguera A, Boeriu E, Poroch V, Elsner F, Mosoiu D (2020). From European Association for Palliative Care Recommendations to a blended, standardized, free-to-access undergraduate curriculum in palliative medicine: the EDUPALL project. J Palliat Med.

[12] A’campo L, Spliethoff-Kamminga N, Macht M, Roos R, Consortium E (2010). Caregiver education in Parkinson’s disease: formative evaluation of a standardized program in seven European countries. J Qual Life Res.

[13] Simons G, Thompson SB, Pasqualini MCS (2006). An innovative education programme for people with Parkinson's disease and their carers. Parkinsonism Related Disord.

[14] Jordan SR, Kluger B, Ayele R, Brungardt A, Hall A, Jones J, Katz M, Miyasaki JM, Lum HD (2020). Optimizing future planning in Parkinson disease: suggestions for a comprehensive roadmap from patients and care partners. J Ann Palliat Med.

[15] Fox S, Cashell A, Kernohan WG, Lynch M, McGlade C, O’Brien T, O’Sullivan SS, Foley MJ, Timmons S (2017). Palliative care for Parkinson’s disease: patient and carer’s perspectives explored through qualitative interview. J Palliative Med.

[16] Sandsdalen T, Grøndahl VA, Hov R, Høye S, Rystedt I, Wilde-Larsson B (2016). Patients’ perceptions of palliative care quality in hospice inpatient care, hospice day care, palliative units in nursing homes, and home care: a cross-sectional study. BMC Palliative Care.

[17] Moens K, Houttekier D, Van den Block L, Harding R, Morin L, Marchetti S (2015). Place of death of people living with Parkinson’s disease: a population-level study in 11 countries. BMC Palliative Care.

[18] Connor K, Cheng E, Siebens HC, Lee ML, Mittman BS, Ganz DA, Vickrey B (2015). Study protocol of “CHAPS”: a randomized controlled trial protocol of care coordination for health promotion and activities in Parkinson’s disease to improve the quality of care for individuals with Parkinson’s disease. BMC Neurol.

[19] van der Eijk M, Faber MJ, Ummels I, Aarts JW, Munneke M, Bloem BR (2012). Patient-centeredness in PD care: development and validation of a patient experience questionnaire. Parkinsonism Related Disord.

[20] McLaughlin D, Hasson F, Kernohan WG, Waldron M, McLaughlin M, Cochrane B, Chambers H (2011). Living and coping with Parkinson’s disease: perceptions of informal carers. J Palliative Med.

[21] Olsson Y, Clarén L, Alvariza A, Årestedt K, Hagell P (2016). Health and social service access among family caregivers of people with Parkinson’s disease. J Parkinsons Dis.

[22] Schrag A, Hovris A, Morley D, Quinn N, Jahanshahi M (2006). Caregiver-burden in Parkinson's disease is closely associated with psychiatric symptoms, falls, and disability. Parkinsonism Related Disord.

[23] Hasson F, Kernohan WG, McLaughlin M, Waldron M, McLaughlin D, Chambers H, Cochrane B (2010). An exploration into the palliative and end-of-life experiences of carers of people with Parkinson’s disease. J Palliative Med.

[24] Goy ER, Carter JH, Ganzini L (2008). Needs and experiences of caregivers for family members dying with Parkinson disease. J Palliat Care.

[25] Sokol LL, Young MJ, Paparian J, Kluger BM, Lum HD, Besbris J, Kramer NM, Lang AE, Espay AJ, Dubaz OM, Miyasaki JM, Matlock DD, Simuni T, Cerf M (2019). Advance care planning in Parkinson’s disease: ethical challenges and future directions. NPJ Parkinsons Dis.

[26] Aoun S, McConigley R, Abernethy A, Currow DC (2010). Caregivers of people with neurodegenerative diseases: profile and unmet needs from a population-based survey in South Australia. J Palliat Med.

[27] Carter J, Lyons K, Lindauer A, Malcom J (2012). Pre-death grief in Parkinson’s caregivers: a pilot survey-based study. J Parkinsonism Related Disord.

[28] Tuck KK, Brod L, Nutt J, Fromme EK (2015). Preferences of patients with Parkinson’s disease for communication about advanced care planning. Am J Hospice Palliative Med.

[29] Walter HA, Seeber AA, Willems DL, De Visser M (2019). The role of palliative care in chronic progressive neurological diseases—a survey amongst neurologists in the Netherlands. Front Neurol.

[30] Miyasaki JM (2016). Treatment of advanced Parkinson disease and related disorders. CONTINUUM: Lifelong Learning in Neurology.

[31] Lum HD, Kluger BM (2020). Palliative Care for Parkinson Disease. J Clinics Geriatric Med.

[32] Robinson MT, Holloway RG. Palliative Care in Neurology. Mayo Clin Proc. 2017;92(10):1592–601. 10.1016/j.mayocp.2017.08.003. 10.1016/j.mayocp.2017.08.003 28982489

[33] Katz M, Goto Y, Kluger BM, Galifianakis NB, Miyasaki JM, Kutner JS, Jones CA, Pantilat SZ (2018). Top ten tips palliative care clinicians should know about Parkinson's disease and related disorders. J Palliat Med.

[34] Robinson MT, Barrett KM (2014). Emerging subspecialties in neurology: neuropalliative care. J Neurol.

[35] Tarolli CG, Holloway RG (2019). Palliative care and Parkinson’s disease: outpatient needs and models of care over the disease trajectory. Ann Palliative Med.

[36] Thomas PA, Kern DE, Hughes MT, Chen BY (2016). Curriculum development for medical education: a six-step approach: JHU press.

[37] Chen BY, Kern DE, Kearns RM, Thomas PA, Hughes MT, Tackett S (2019). From modules to MOOCs: application of the six-step approach to online curriculum development for medical education. J Acad Med.

[38] Hoy MB (2014). MOOCs 101: an introduction to massive open online courses. J Med Ref Serv Quarterly.

[39] Goldberg LR, Crocombe LA (2017). Advances in medical education and practice: role of massive open online courses. J Adv Med Educ Pract.

[40] Mehta NB, Hull AL, Young JB, Stoller JK (2013). Just imagine: new paradigms for medical education. J Acad Med.

[41] Subhi Y, Andresen K, Bojsen SR, Nilsson PM, Konge L (2014). Massive open online courses are relevant for postgraduate medical training. Dan Med J.

[42] Koch S, Hägglund M (2017). Mutual learning and exchange of health informatics experiences from around the world-evaluation of a massive open online course in eHealth. Stud Health Technol Inform.

