# Supplementary material for: Education on palliative care for Parkinson patients: development of the “Best care for people with late-stage Parkinson’s disease” curriculum toolkit
Source: BMC Med Educ. 2021 Oct 25;21:538. doi: 10.1186/s12909-021-02964-6 (PMC8547059; doi:10.1186/s12909-021-02964-6)
Supplement: Supplementary file 1 — Additional file 1. [file 12909_2021_2964_MOESM1_ESM.docx]

# The PD_Pal Curriculum Toolkit

| **Lesson Plan** | |
| --- | --- |
| **Teaching Unit** | Parkinson’s Disease |
| **Title** | **Week 1: Parkinson’s Disease** |
| **Learning Objectives** | Getting familiar with Parkinson’s disease in terms of epidemiology and prevalence, known causes including genetics, environmental and their interactions, how diagnosis is made and communicated and which are the main symptoms.  Understanding the impact of PD to patients and their caregivers and how the early and timely integration of palliative care can help deal with it. |
| **Summary &**  **scheduling** | Part 1: Facts, cause, diagnosis and symptoms  Part 2: Expected impact of diagnosis, prognosis and importance of timely integration of Palliative Care |
| **Learning Content A - Attitude K - Knowledge S - Skills** | Attitude: The clinicians are encouraged to be respectful towards patients and caregivers need for information. To encourage patients and caregivers ask the questions.  Knowledge: Participants know the basics of PD, specifically:   - What PD is - The factors that modify the risk of developing PD - The pathogenetic mechanisms that lead to the degeneration of PD - The clinical features of the disorder - The presentation and diagnosis of PD - What is the prognosis of the disease and the timing of introducing Palliative Care   Skills: Clinicians are able to recognise PD and its complex symptoms and discuss disease management with their patients and caregivers. |
| **Teaching Methods** | - Presentation (slides) - Video component - Reading materials |
| **Literature** | - Tysnes OB, Storstein A. Epidemiology of Parkinson's disease. J Neural Transm (Vienna). 2017;124(8):901–905. - Miller IN, Cronin-Golomb A. Gender differences in Parkinson's disease: clinical characteristics and cognition. Mov Disord. 2010;25(16):2695–2703. - Billingsley KJ, Bandres-Ciga S, Saez-Atienzar S, Singleton AB. Genetic risk factors in Parkinson's disease. Cell Tissue Res. 2018;373(1):9–20. - Hernandez DG, Reed X, Singleton AB. Genetics in Parkinson disease: Mendelian versus non-Mendelian inheritance. J Neurochem. 2016;139 Suppl 1(Suppl 1):59–74. - Ball N, Teo WP, Chandra S, Chapman J. Parkinson's Disease and the Environment. Front Neurol. 2019;10:218. Published 2019 Mar 19. - Surmeier DJ. Determinants of dopaminergic neuron loss in Parkinson's disease. FEBS J. 2018;285(19):3657–3668. - Cherubini M, Wade-Martins R. Convergent pathways in Parkinson's disease. Cell Tissue Res. 2018;373(1):79–90. - Marsili L, Rizzo G, Colosimo C. Diagnostic Criteria for Parkinson's Disease: From James Parkinson to the Concept of Prodromal Disease. Front Neurol. 2018;9:156. Published 2018 Mar 23. - Vu TC, Nutt JG, Holford NH. Progression of motor and nonmotor features of Parkinson's disease and their response to treatment. Br J Clin Pharmacol. 2012;74(2):267–283. - Sarafis P, Tsounis A, Malliarou M, Lahana E. Disclosing the truth: a dilemma between instilling hope and respecting patient autonomy in everyday clinical practice. Glob J Health Sci. 2013;6(2):128–137. Published 2013 Dec 20. - van der Steen JT, Lennaerts H, Hommel D, et al. Dementia and Parkinson's Disease: Similar and Divergent Challenges in Providing Palliative Care. Front Neurol. 2019;10:54. Published 2019 Mar 11 |
| **Assessment** | - Topic related Discussion/Exercise - Self-Assessment (20 multiple choice questions) |

| **Lesson Plan** | |
| --- | --- |
| **Teaching Unit** | Palliative care principles and concepts |
| **Title** | **Week 2: Palliative care principles and concepts** |
| **Learning Objectives** | Comprehending the palliative care principles.  Get introduced to the multi-dimensionality of human-being and the complexity of accepting death and non-ideal outcomes.  Understanding the dynamic involvement of palliative care throughout the illness.  Get familiar with patient-family oriented care management competencies.  Taking care of oneself and multidisciplinary team. |
| **Summary &**  **scheduling** | Part 1- Palliative care philosophy, policy and definitions. Basic understanding of complex symptoms assessment and management and shared decision making.  Part 2- Importance of Self-Care and multidisciplinary team. |
| **Learning Content A - Attitude K - Knowledge S - Skills** | Attitude: Participants will see palliative care in terms of the comfort it offers to patients and caregivers. Understand the importance of the unit of care approach.  Knowledge: Participants know the basics of palliative care, specifically:   - What palliative care is - The dynamic involvement of palliative care throughout the illness - The Palliative care principles - The multi-dimensionality of human-being - The importance of self-care   Skills: Participants will be able to better handle issues related to the complexity of accepting death and non-ideal outcomes. |
| **Teaching Methods** | - Presentation (slides) - Video component - Self-Assessment - Reading materials |
| **Literature** | - Porter, R. (1999). The Greatest Benefit to the Mankind: A Medical History of Humanity from Antiquity to the Present. Los Angeles: Fontana Press. - Vanderpool, H. Y. (2015). Palliative Care: The 400-year Quest for a Good Death. North Carolina: McFahrland & Company, Inc, Publishers. - World Health Organization. (2002). Palliative care. <http://www.who.int/cancer/palliative/definition/en/> - Oliver, D.J. et al. (2016). A consensus review on the development of palliative care for patients with chronic and progressive neurological disease. Eur J Neurol. 23(1), 30-38. - Clark, D. (2015). Public debate begins in Scotland on future of NHS. BMJ, 351, h4266. - Etkind, S. N. et al. (2017). How many people will need palliative care in 2040? Past trends, future projections and implications for services. BMC Med, 15, 102. - WPCA (2014). Global Atlas of Palliative Care at the End of Life. In: WHO (ed.). - WHO (2014). Strengthening of Palliative Care as a Component of Integrated Treatment throughout the Life Course. Journal of Pain & Palliative Care Pharmacotherapy, 28, 130-134. - Powell, R. A. et al. (2015). Putting palliative care on the global health agenda. The Lancet Oncology, 16, 131-133. - UNICEF, W. (2018). Astana Declaration. Astana, Kazakhstan. - Line, D. (2015). Quality of Death Index. <https://eiuperspectives.economist.com/healthcare/2015-quality-death-index> - IAHPC (2019). Palliative Care Definition. <https://hospicecare.com/what-we-do/projects/consensus-based-definition-of-palliative-care/definition/> - WHO (2017). Ten Facts on Palliative Care. <https://www.who.int/features/factfiles/palliative-care/en/> - Becker, E. (1973). The Denial of Death. New York: Simon & Schuster. - Dor-Ziderman, Y., Lutz, A. & Goldstein, A. (2019). Prediction-based neural mechanisms for shielding the self from existential threat. Neurolmage, Volume 202. - Department of Health (2012). End of Life Care Strategy: Fourth Annual Report. [https://webarchive.nationalarchives.gov.uk/20130104174405/http://www.dh.gov.uk/health/2012/10/end-of-life-care-fourth/](https://webarchive.nationalarchives.gov.uk/20130104174405/http:/www.dh.gov.uk/health/2012/10/end-of-life-care-fourth/) - Paal, P. et al. (2019). Postgraduate palliative care education for all healthcare providers in Europe: Results from an EAPC survey. Palliative and Supportive Care, 1-12. - Boyd, M. (2016). My Life, My Death: The Voices of Palliative Patients, Informal Caregivers and Health Care Providers. Journal of Pain and Symptom Management, 52, E95-E96. - BMJ (2017). Palliative Care from Diagnosis to death. <https://youtu.be/vS7ueV0ui5U> |
| **Assessment** | - Topic related Discussion/Exercise - Self-Assessment (20 multiple choice questions) |

| **Lesson Plan** | |
| --- | --- |
| **Teaching Unit** | Parkinson’s Disease and its management |
| **Title** | **Week 3: Parkinson’s Disease and its management** |
| **Learning Objectives** | Be informed about the available pharmacological treatments for PD.  Be informed about the surgical options that exist for the advanced Parkinson’s Disease.  Comprehend the importance of multi-professional, interdisciplinary and continuous management of PD. |
| **Summary &**  **scheduling** | Part 1: drug therapy and surgical options for PD.  Part 2: information about complementary therapies and occupational therapy, introduction to multi-professional, interdisciplinary approaches for the management of PD. |
| **Learning Content A - Attitude K - Knowledge S - Skills** | Attitude:  To engage patients and caregivers into their care management. All participants will recognize the importance of multi-professional, interdisciplinary approaches for the management of PD.  Knowledge: Participants know the basics of PD management options, specifically:   - Which are the therapeutic options for Parkinson’s Disease (PD) - The indications, effectiveness and adverse effects of drug and surgery treatments - The role of non-pharmacological therapies and of allied professionals   Skills: Participants will be able to direct patients and caregivers to the best sources of information. They will also be able to provide practical aids and solutions to keep patients independent. |
| **Teaching Methods** | - Presentation (slides) - Video component - Reading materials |
| **Literature** | - Aragon A. and Kings J. (2018). Occupational therapy for people with Parkinson’s (Second edition). London: Copyright © Royal College of Occupational Therapists. - Cianci H. (2001). Activities of Daily Living: Practical Pointers for Parkinson’s Disease (Third edition). Miami: National Parkinson Foundation. - Dietrichs E, Odin P. Algorithms for the treatment of motor problems in Parkinson's disease. Acta Neurol Scand. 2017;136(5):378–385. - Radder DLM, de Vries NM, Riksen NP, et al. Multidisciplinary care for people with Parkinson's disease: the new kids on the block!. Expert Rev Neurother. 2019;19(2):145–157. - Rabin ML, Stevens-Haas C, Havrilla E, et al. Complementary Therapies for Parkinson's Disease: What's Promoted, Rationale, Potential Risks and Benefits. Mov Disord Clin Pract. 2015;2(3):205–212. - Hauser, RA. (2020). Parkinson Disease Treatment & Management. (Access on 14.04.2020). Medscape <https://emedicine.medscape.com/article/1831191-treatment#d1> |
| **Assessment** | - Topic related Discussion/Exercise - Self-Assessment (20 multiple choice questions) |

| **Lesson Plan** | |
| --- | --- |
| **Teaching Unit** | Interdisciplinary approaches for the management of PD |
| **Title** | **Week 4: Interdisciplinary approaches for the management of PD** |
| **Learning Objectives** | Understand the concepts of multidisciplinary and interdisciplinary approaches and their difference.  Identify the different healthcare professionals involved in the management of patients with Parkinson’s disease and their roles.  Learn how to design individual care plans by taking the needs and preferences of the patient and caregiver into account. |
| **Summary &**  **scheduling** | How to design care plans accordingly to patients’ and families’ wishes integrating multi-professional and interdisciplinary approaches (3 hours). |
| **Learning Content A - Attitude K - Knowledge S - Skills** | Attitude:  Prioritize patients and caregivers needs and design and implement personalized, interdisciplinary management and care plans based on shared decision making. Acknowledge the role of allied professionals.  Knowledge: Participants know the basics of interdisciplinary care, specifically:   - multi-professional, coordinated care - the different healthcare professionals involved in patients with Parkinson’s disease - when to involve which healthcare professional according to the care needs of the patient - how to design individual care plans by taking the wishes of the patient and caregiver into account   Skills: Participants will be able to understand the competencies interplay and the professional limits, as well as who/what is the best source to give/get sufficient solutions and answers for the patients’ needs. Adopt a methodology for providing interdisciplinary care based on patients’ and caregivers’ needs. |
| **Teaching Methods** | - Presentation (slides) - Video component - Reading materials |
| **Literature** | - Brinkman-Stoppelenburg, A., Rietjens, J. A., & Van der Heide, A. (2014). The effects of advance care planning on end-of-life care: a systematic review. Palliative medicine, 28(8), 1000-1025. - Bruera, E., Kuehn, N., Miller, M. J., Selmser, P., & Macmillan, K. (1991). The Edmonton Symptom Assessment System (ESAS): A Simple Method for the Assessment of Palliative Care Patients. Journal of Palliative Care, 7(2), 6-9. doi:10.1177/082585979100700202 - Fox, S., Azman, A., & Timmons, S. (2020). Palliative care needs in Parkinson’s disease: focus on anticipatory grief in family carers. Annals of Palliative Medicine, 9(Supplement 1), 34-43. - Fox, S., Cashell, A., Kernohan, W. G., Lynch, M., McGlade, C., O’Brien, T., . . . Timmons, S. (2017). Palliative care for Parkinson’s disease: Patient and carer’s perspectives explored through qualitative interview. Palliative medicine, 31(7), 634-641. - Giladi, N., Manor, Y., Hilel, A., & Gurevich, T. (2014). Interdisciplinary Teamwork for the Treatment of People with Parkinson’s Disease and Their Families. Current Neurology and Neuroscience Reports, 14(11), 493. doi:10.1007/s11910-014-0493-1 - Giles, S., & Miyasaki, J. (2009). Palliative stage Parkinson’s disease: patient and family experiences of health-care services. Palliative medicine, 23(2), 120-125. - Hudson, P. L., Toye, C., & Kristjanson, L. J. (2006). Would people with Parkinson's disease benefit from palliative care? Palliative medicine, 20(2), 87-94. - Irish Palliative Care in Parkinson’s Disease Group. (2016). Palliative care in People with Parkinson’s disease: Guidelines for professional healthcare workers on the assessment and management of palliative care needs in Parkinson’s disease and related Parkinsonian syndromes. In: University College Cork Cork. - Kalf, J., de Swart, B., Bonnier, M., Hofman, M., Kanters, J., Kocken, J., . . . Munneke, M. (2011). Guidelines for speech-language therapy in Parkinson's disease. Nijmegen, The Netherlands/Miami, FL: ParkinsonNet/NPF. - Keus, S., Munneke, M., Graziano, M., Paltamaa, J., Pelosin, E., Domingos, J., . . . Struiksma, C. (2014). European physiotherapy guideline for Parkinson’s disease. The Netherlands: KNGF/ParkinsonNet. - Lennaerts, H., Groot, M., Rood, B., Gilissen, K., Tulp, H., van Wensen, E., . . . Bloem, B. R. (2017). A Guideline for Parkinson’s Disease Nurse Specialists, with Recommendations for Clinical Practice. Journal of Parkinson's Disease, 7, 749-754. doi:10.3233/JPD-171195 - McLaughlin, D., Hasson, F., Kernohan, W. G., Waldron, M., McLaughlin, M., Cochrane, B., & Chambers, H. (2011). Living and coping with Parkinson’s disease: perceptions of informal carers. Palliative medicine, 25(2), 177-182. - Miyasaki, J. M., & Kluger, B. (2015). Palliative care for Parkinson’s disease: has the time come? Current Neurology and Neuroscience Reports, 15(5), 26. - Miyasaki, J. M., Long, J., Mancini, D., Moro, E., Fox, S., Lang, A., . . . Arshinoff, R. (2012). Palliative care for advanced Parkinson disease: an interdisciplinary clinic and new scale, the ESAS-PD. Parkinsonism & related disorders, 18, S6-S9. - Radder, D. L. M., de Vries, N. M., Riksen, N. P., Diamond, S. J., Gross, D., Gold, D. R., . . . Bloem, B. R. (2019). Multidisciplinary care for people with Parkinson’s disease: the new kids on the block! Expert Review of Neurotherapeutics, 19(2), 145-157. doi:10.1080/14737175.2019.1561285 - Sturkenboom, I., Thijssen, M., Gons-van Elsacker, J., Jansen, I., Maasdam, A., Schulten, M., . . . Munneke, M. (2011). Guidelines for occupational therapy in Parkinson's disease rehabilitation. Nijmengen, The Netherlands/Miami (FL), USA: ParkinsonNet/NPF. Heruntergeladen von http://www.parkinsonnet. info/media/14820461/ot_guidelines_final-npf__3_. pdf am, 3, 2016. - van Asseldonk, M., Dicke, H., van den Beemt, B., van den Berg, D., ter Borg, S., Duin, G., . . . van Harten, B. (2012). Dietetic guideline for Parkinson’s. - van der Marck, M. A., & Bloem, B. R. (2014). How to organize multispecialty care for patients with Parkinson's disease. Parkinsonism & related disorders, 20, S167-S173. - van der Marck, M. A., Kalf, J. G., Sturkenboom, I. H. W. M., Nijkrake, M. J., Munneke, M., & Bloem, B. R. (2009). Multidisciplinary care for patients with Parkinson's disease. Parkinsonism & related disorders, 15, S219-S223. doi:https://doi.org/10.1016/S1353-8020(09)70819-3 - WHO. WHO Definition of palliative care Retrieved from https://www.who.int/cancer/palliative/definition/en/ |
| **Assessment** | - Topic related Discussion/Exercise - Self-Assessment (20 multiple choice questions) |

| **Lesson Plan** | |
| --- | --- |
| **Teaching Unit** | Getting on with life (Living with Parkinson’s) |
| **Title** | **Week 5: Getting on with life** (**Living with Parkinson’s)** |
| **Learning Objectives** | Understand the impact of PD in common activities of daily life.  Learning practical tips that will help patients and caregivers improve their quality of life. |
| **Summary &**  **scheduling** | Parkinson’s disease (PD) may impact day to day life and make some daily routines more burdensome. Having a positive attitude to life and maintaining an active daily routine adapting daily activities as much as possible is vital for the management of PD in the long term. |
| **Learning Content A - Attitude K - Knowledge S - Skills** | Attitude:  Keeping a positive attitude to life. Remaining optimistic. Acknowledging the role and needs of informal caregivers.  Knowledge: Participants are informed about:   - Sleep disturbances - Emotional health - Cognitive decline - Economic and social burden   Skills: Participants will be provided with practical advice on how to cope with aspects of daily living (getting a good night’s sleep, cognitive training etc.) |
| **Teaching Methods** | - Presentation (slides) - Video component - Reading materials |
| **Literature** | - InfoPark Infosheets: QLRT 2000-00303 (2001-2004, European Commission) - Information, health and social needs of older, disabled people (Parkinson's disease) and their carers / InfoPark. - Miyasaki, J. M., & Kluger, B. (2015). Palliative care for Parkinson’s disease: has the time come?. Current neurology and neuroscience reports, 15(5), 26. - McLaughlin et al. (2010). Living and coping with Parkinson’sdisease: Perceptions of informal carers. Palliative Medicine, 25(2), 177–182. - Goy, E.R., Boling, A., Carter, J. (2015). Identifying Predictors of Hospice Eligibility in Patients With Parkinson Disease. American Journal of Hospice& Palliative Medicine, 32(1), 29-33. - Strupp, J., Kunde, A., Galushko, M.,Voltz, R., Golla, H. (2017). Severely Affected by Parkinson Disease:The Patient’s View and Implicationsfor Palliative Care. American Journal of Hospice& Palliative Medicine, 1-7. - Fereshtehnejad, S.-M. (2016). Strategies to maintain quality of life among people with Parkinson’s disease: what works?. Neurodegenerative Disease Management, 6(5), 399–415. - Lim, S.-Y., Tan, A. H., Fox, S. H, Evans, A. H., Low, S. C. (2017). Integrating Patient Concerns into Parkinson’sDisease Management. Curr Neurol Neurosci Rep, 17:3. - Titova, N., Chaudhuri, R. K. (2017). Palliative Care and NonmotorSymptoms in Parkinson’s Diseaseand Parkinsonism. International Review of Neurobiology,134, 1239-55. - Katz, M., Goto, Y., Kluger, B. M. (2018). Top Ten Tips Palliative Care Clinicians Should Know About Parkinson’s Disease and Related Disorders. Journal of Palliative Medicine, 21(10):1507-1517. |
| **Assessment** | - Topic related Discussion/Exercise - Self-Assessment (20 multiple choice questions) |

| **Lesson Plan** | |
| --- | --- |
| **Teaching Unit** | Providing care for caregivers |
| **Title** | **Week 6: Providing care for caregivers** |
| **Learning Objectives** | Comprehend what caregiving entails.  Understand the rights and needs of caregivers.  Be educated in basic caring skills.  Become aware of interventions aiming to reduce the burden and distress of the caregiver.  Understand the role of support groups. |
| **Summary &**  **scheduling** | Parkinson’s disease (PD) may impact caregivers’ day to day life and make some daily routines more burdensome. Having a positive attitude to life and maintaining an active daily routine adapting daily activities as much as possible is vital for the management of PD in the long term (3 hours). |
| **Learning Content A - Attitude K - Knowledge S - Skills** | Attitude: Become aware of stress factors and the fact that caregivers’ emotions need special attention. Grasping the importance of looking after oneself as a carer: avoiding burn out, accepting outside help and sharing the caring tasks.  Knowledge: Participants will know:   - Caregiver and caregiving concepts and definitions - The rights and needs of caregivers - the role of support groups that may ease the caregiver burden   Skills: Participants will be able to recognise and address caregivers’ burden. Practical advice and tips on basic caring skills to prevent burn out and reduce the distress of the caregiver. |
| **Teaching Methods** | - Presentation (slides) - Video component - Reading materials |
| **Literature** | - Boersma, I., Jones, J., Coughlan, C., Carter, J., Bekelman, D., Miyasaki, J., ... & Kluger, B. (2017). Palliative care and Parkinson's disease: caregiver perspectives. Journal of Palliative Medicine, 20(9), 930-938. - Lokk, J., & Delbari, A. (2012). Clinical aspects of palliative care in advanced Parkinson’s disease. BMC palliative care, 11(1), 20. - Miyasaki, J. M., & Kluger, B. (2015). Palliative care for Parkinson’s disease: has the time come? Current neurology and neuroscience reports, 15(5), 26. - Abernethy, A. P., Currow, D. C., Fazekas, B. S., Luszcz, M. A., Wheeler, J. L., & Kuchibhatla, M. (2008). Specialized palliative care services are associated with improved short-and long-term caregiver outcomes. Supportive Care in Cancer, 16(6), 585-597. - Bédard, M., Molloy, D. W., Squire, L., Dubois, S., Lever, J. A., & O'Donnell, M. (2001). The Zarit Burden Interview: a new short version and screening version. The Gerontologist, 41(5), 652-657. - Zarit, S. H., Reever, K. E., & Bach-Peterson, J. (1980). Relatives of the impaired elderly: correlates of feelings of burden. The gerontologist, 20(6), 649-655. - Martínez‐Martín, P., Forjaz, M. J., Frades‐Payo, B., Rusinol, A. B., Fernández‐García, J. M., Benito‐León, J., ... & Catalán, M. J. (2007). Caregiver burden in Parkinson's disease. Movement disorders, 22(7), 924-931. - Martinez-Martin, P., Rodriguez-Blazquez, C., Forjaz, M. J., Frades-Payo, B., Agüera-Ortiz, L., Weintraub, D., ... & Chaudhuri, K. R. (2015). Neuropsychiatric symptoms and caregiver's burden in Parkinson's disease. Parkinsonism & Related Disorders, 21(6), 629-634. - Macchi, Z. A., Koljack, C. E., Miyasaki, J. M., Katz, M., Galifianakis, N., Prizer, L. P., ... & Kluger, B. M. (2019). Patient and caregiver characteristics associated with caregiver burden in Parkinson's disease: a palliative care approach. Annals of palliative medicine. - Prizer, L. P., Kluger, B. M., Sillau, S., Katz, M., Galifianakis, N., & Miyasaki, J. M. (2019). Correlates of spiritual wellbeing in persons living with Parkinson disease. Annals of Palliative Medicine, 9(Suppl 1), S16-S23. - Fox, S., Azman, A., & Timmons, S. (2020). Palliative care needs in Parkinson’s disease: focus on anticipatory grief in family carers. Annals of Palliative Medicine, 9 (Supplement 1), 34-43. - InfoPark Infosheets: QLRT 2000-00303 (2001-2004, European Commission) - Information, health and social needs of older, disabled people (Parkinson's disease) and their carers. - EduPark Infosheets; QLRT 2001-02674 (2003-2005, European Commission, 258 587 Eur) - Patient education in Parkinson’s disease). |
| **Assessment** | - Topic related Discussion/Exercise - Self-Assessment (20 multiple choice questions) |

| **Lesson Plan** | |
| --- | --- |
| **Teaching Unit** | Advance Care Planning and Advance Directives |
| **Title** | **Week 7: Advance Care Planning and Advance Directives** |
| **Learning Objectives** | Be able to identify and describe the different “last decisions” in late stage PD.  Have basic knowledge on how to make a will.  Know basics on how to deal with power of attorneys.  Being part of science: participating in clinical studies and the “brain bank” initiative.  Have some basic knowledge on the most important research activities in late stage PD. |
| **Summary &**  **scheduling** | Part 1: Getting to know Advance Care Directives and what Planning includes  Part 2: Relevant Research activities |
| **Learning Content A - Attitude K - Knowledge S - Skills** | Attitude: Participants will get familiar with the whole complex subject of Advanced Care Planning (ACP) and of the, most times, challenging, decisions that it entails. Patients are encouraged to participate in clinical studies and even donate cells and tissues.  Knowledge: Participants will:   - learn what ACP is and what it includes - get some basic knowledge on prognostics in PD - be informed about the Power of Attorneys and other procedures - be informed about the importance of the research concerning late stage PD   Skills: Participants are able to explain ACP concepts and its benefits. They are also able to plan advance care according to patients’ needs and preferences. |
| **Teaching Methods** | - Presentation (slides) - Video component - Reading materials |
| **Literature** | - Balzer-Geldsetzer M, Ferreira J, Odin P, Bloem BR, Meissner WG, Lorenzl S, Wittenberg M, Dodel R, Schrag A. Study protocol: Care of Late-Stage Parkinsonism (CLaSP): a longitudinal cohort study. BMC Neurology 2018, 5; 18(1): 185. - Bower JH, Maraganore DM, McDonnell SK, Rocca WA. Incidence and distribution of Parkinsonism in Olmsted Coutry, Minnesota, 1976-1990. Neurology 1999 (52): 1214-1220. - DZNE Brain Bank (ed.) Biomaterial Bank of Postmortem Brain Tissue for the Resarch on Neurodegenerative Diseases, https://www.dzne.de/forschung/brain-bank/ (30.03.2020) - Ebke M, Koch A, Dillen K, Becker I, Voltz R, Golla H. (2018) The “Surprise Question” in Neurorehabilitation—Prognosis Estimation by Neurologist and Palliative Care Physician; a Longitudinal, Prospective, Observational Study. Frontiers in Neurology, 2018, 9:792. - NICE guideline (ed.) [NG 71]. Parkinson´s Disease in adults, 2017. https://www.nice.org.uk/guidance/ng71/chapter/Recommendations#palliative-care (01.04.2020). - Oliver D, Borasio GD, Caraceni A, de Visser M, Grisold W, Lorenzl S, Veronese S, Voltz R. Palliative care in chronic and progressive neurological disease: summary of a consensus review. European Journal of Palliative Care 2016; 23(5): 232-235. - Parkinsons.org.uk (ed.): preparing for end of life. https://www.parkinsons.org.uk/information-and-support/preparing-end-life (05.04.2020). - https://mrc.ukri.org/research/facilities-and-resources-for-researchers/brain-banks/about-the-uk-brain-banks-network/ (24.03. 2020). - WHO (ed.). State of the world's nursing 2020: investing in education, jobs and leadership. Geneva: World Health Organization; 2020. - Seppi K, Ray Chaudhuri K, Coelho M, Fox SH, Katzenschlager R, Perez Lloret S, Weintraub D, Sampaio C; and the collaborators of the Parkinson's Disease Update on Non-Motor Symptoms Study Group on behalf of the Movement Disorders Society Evidence-Based Medicine Committee. Update on treatments for nonmotor symptoms of Parkinson's disease-an evidence-based medicine review. Mov Disord. 2019 Jan 17. doi: 10. 1002/mds. 27602. |
| **Assessment** | - Topic related Discussion/Exercise - Self-Assessment (20 multiple choice questions) |

| **Lesson Plan** | |
| --- | --- |
| **Teaching Unit** | Advanced Parkinson`s Disease, Death and Dying |
| **Title** | **Week 8: Advanced Parkinson`s Disease, Death and Dying** |
| **Learning Objectives** | Be able to recognise the advanced stage PD and start or increase the intensity of palliative care.  Understand the difficulties of the advanced stage.  Understand and accept the end-of-life decision-making process.  Be aware of alternative settings and different options for the advanced stage. |
| **Summary &**  **scheduling** | Helping to comprehend what advanced stage PD means and what additional difficulties and complexities it entails (3 hours). |
| **Learning Content A - Attitude K - Knowledge S - Skills** | Attitude: Participants will comprehend that the patients and caregivers might need extra support in the advanced stage coping with the disease.  Knowledge: Participants will get to know:   - Global facts about the advanced stage of PD - The meaning of falling out of care - The gender gap - Prognostication in PD - Symptoms and indications of advanced stage of PD - Triggers and indicators for referral to specialist palliative care   Skills: Participants are able to coordinate and manage an informed and structured decision-making process, including referral to palliative care in different care settings. |
| **Teaching Methods** | - Presentation (slides) - Video component - Reading materials |
| **Literature** | - Baldereschi M, DiCarlo A, RoccaWA, Vanni P, Maggi S, Perissinotto E, Grigoletto F, Amaducci L, Inzitari D. (2000): Parkinson’s disease and parkinsonism in a longitudinal study: Two-fold higher incidence in men. ILSA Working Group. Italian Longitudinal Study on Aging. Neurology 9, 1358-1363. - Bükki J, Nübling G, Lorenzl S. (2014): Managing Advanced Progressive Supranuclear Palsy and Corticobasal Degeneration in a Palliative Care Unit: Admission Triggers and Outcomes. American Journal of Hospice and Palliative Medicine. https://doi.org/10.1177/1049909114565110. - Cerri S, Mus L, Blandine F. (2019): Parkinson’s Disease in Women and Men: What’s the Difference? Journal of Parkinson’s Disease 9 (2019) 501–515. doi: 10.3233/JPD-191683. - Dahodwala N, Shah K, He Y, Wu SS, Schmidt P,Cubillos F, Willis AW (2018): Sex disparities in access to caregiving in Parkinson disease. Neurology 90, 48-e54.  doi: 10.1212/WNL.0000000000004764 . - Dexter DT, Jenner P (2013): Parkinson disease: From pathology to molecular disease mechanisms. Free Radic Biol Med 62, 132-144, doi: 10.1016/j.freeradbiomed.2013.01.018. - Enders D, Balzer-Geldsetzer M, Riedel O et al. (2017): “Prevalence, duration and severity of Parkinson’s disease in Germany: a combined meta-analysis from literature data and outpatient samples,” Europ Neurol, 78(3-4):128–136. doi: 10.1159/000477165. - Goy E R, Bohlig A, Carter J. Ganzini L. (2013): Identifying Predictors of Hospice Eligibility in Patients with Parkinson Disease. American Journal of Hospice and Palliative Medicine. ttps://doi.org/10.1177/1049909113502119 - Gries CJ, Engelberg RA, Erin K. Kross, Doug Zatzick, Elizabeth L. Nielsen, Lois Downey J, Randall Curtis, Predictors of Symptoms of Posttraumatic Stress and Depression in Family Members After Patient Death in the ICU, Chest, Volume 137, Issue 2, 2010, 280-287, https://doi.org/10.1378/chest.09-1291. - Lokk J, Delbari A. Clinical aspects of palliative care in advanced Parkinson's disease. BMC Palliat Care 2012 ,11:20. Published 2012 Oct 25. doi:10.1186/1472-684X-11-20. - Lorenzl S, Nübling G, Perrar KM, Voltz R. (2013): Palliative treatment of Chronic neurological disorders. In: James L, Bernat H, Beresford R. (ed.) (2013): Ethical and Legal Issues in Neurology 118: 133-139. - Nübling S, Schuberth M, Feldmer K, Giese A, Holdt L M, Teupser D, Lorenzl S. (2017): Cathepsin S increases tau oligomer formation through limited cleavage, but only IL-6, not cathespin S serum levels correlate with disease severity in the neurodegenerative tauopathy progressive supranuclear palsy. Experimental Brain Research 235: 2407-2412. - Oliver DJ, Borasio GD, Caraceni A, et al. A consensus review on the development of palliative care for patients with chronic and progressive neurological disease. Eur J Neurol. 2016;23(1):30‐38. doi:10.1111/ene.12889. - Oliver DJ, Veronese S. Specialist palliative care for Parkinson`s Disease. Ann Palliat Med 2020;9(Suppl 1):52-62. http://dx.doi.org/10.21037/apm.2019.12.01. - Papapetropoulos S, Mash D C (2005): Psychotic Symptoms in Parkinson`s disease. Journal of Neurol 252:753-764. - Petrinec AB, Mazanec PM, Burant CJ, Hoffer A, Daly BJ. (2015): Coping Strategies and Posttraumatic Stress Symptoms in Post-ICU Family Decision Makers. Crit Care Med.;43(6):1205‐1212. doi:10.1097/CCM.0000000000000934. - Snell K, Pennington S, Lee M, Walker R. (2009): The place of death in Parkinson`s disease. Age and Ageing 38(5): 617-619. doi: 10.1093/ageing/afp123. - Lim SY, Tan AH, Ahmad-Annuar A, Klein C, Tan LCS, Rosales RL, Bhidayasiri R, Wu YR, Shang HF, Evans AH, Pal PK, Hattori N, Tan CT, Jeon B, Tan EK, Lang AE. Parkinson's disease in the Western Pacific Region. Lancet Neurol. 2019 Sep;18(9):865-879. doi: 10.1016/S1474-4422(19)30195-4. - Ben-Joseph A, Marshall CR, Lees AJ, Noyce AJ. Ethnic Variation in the Manifestation of Parkinson's Disease: A Narrative Review. J Parkinsons Dis. 2020;10(1):31-45. doi: 10.3233/JPD-191763. |
| **Assessment** | - Topic related Discussion/Exercise - Self-Assessment (20 multiple choice questions) |

| **Lesson Plan** | |
| --- | --- |
| **Teaching Unit** | Managing common symptoms in Late Stage PD |
| **Title** | **Week 9: Managing common symptoms in Late Stage PD** |
| **Learning Objectives** | Understand the clinical aspects of specialist palliative care in Parkinson’s disease.  Be able to recognize, assess and manage common symptoms in late stage PD.  Understand the social aspects of eating.  Swallowing problems in the advanced stages of the disease.  Comprehend when a feeding tube is indicated and what it entails.  Be aware of speech therapeutics options towards eating. |
| **Summary &**  **scheduling** | Part 1: Best palliative care model to provide the patient with comfort and support. A summary of the palliative care management issues and palliative care management options of late stage PD patients.  Part 2: Nutrition related issues and problems which may arise when caring for somebody suffering from late stage Parkinson`s disease and the different management options including feeding tubes. |
| **Learning Content A - Attitude K - Knowledge S - Skills** | Attitude: In advanced PD patients, the focus of treatment shifts to treating the predominant non-motor symptoms and having a more supportive and comforting nature. Participants will understand the social dimension of nutrition beyond medical decision making.  Knowledge: Participants will get to know how late stage symptoms are managed. Specifically:   - Motor symptoms and motoric complications including rigidity and mobility - Autonomic symptoms including pain - Psychiatric complications including depression, hallucinations, cognitive decline - Hypoactive delirium - Breathing dysregulation - Dopaminergic crisis - Obstipation and severe vomiting - The social importance of food - Nutrition and Hydration in the advanced stage of PD - Swallowing problems - Ethical and social implication of feeding tubes (e.g.) PEG-tubes - Hygiene and managing the side effects   Skills: Being able to treat motor complications in late-stage PD needs to increase the time with a view to decreasing dyskinesias and decreasing the occurrence of motor and non-motor off times. Practical skills also include palliative sedation (timeframe, indications etc.) and availability of scheduled analgesics & analgesics on demand (PRN). Moreover, how to manage specific emergencies which might occur in the final phase, for example, the dopaminergic crisis and vomiting. Be able to recognise and deal with swallowing problems. Understand what the installation of a gastric tube means for a patient, how care should be provided, and which are the alternatives. |
| **Teaching Methods** | - Presentation (slides) - Video component - Reading materials |
| **Literature** | - Schrag A, Hommel ALAJ, Lorenzl S, Meissner WG, Odin P, Coelho M, Bloem BR, Dodel R; CLaSP consortium. The late stage of Parkinson's -results of a large multinational study on motor and non-motor complications. Parkinsonism Relat Disord. 2020 Jun;75:91-96. doi: 10.1016/j.parkreldis.2020.05.016. - Hommel ALAJ, Meinders MJ, Weerkamp NJ, Richinger C, Schmotz C, Lorenzl S, Dodel R, Coelho M, Ferreira JJ, Tison F, Boraud T, Meissner WG, Rosqvist K, Timpka J, Odin P, Wittenberg M, Bloem BR, Koopmans RT, Schragand A; CLaSP consortium. Optimizing Treatment in Undertreated Late-Stage Parkinsonism: A Pragmatic Randomized Trial. J Parkinsons Dis. 2020;10(3):1171-1184. doi: 10.3233/JPD-202033. - Hommel ALAJ, Meinders MJ, Lorenzl S, Dodel R, Coelho M, Ferreira JJ, Laurens B, Spampinato U, Meissner W, Rosqvist K, Timpka J, Odin P, Wittenberg M, Bloem PhD BR, Koopmans RT, Schrag A; Care of Late‐Stage Parkinsonism Consortium. The Prevalence and Determinants of Neuropsychiatric Symptoms in Late-Stage Parkinsonism. Mov Disord Clin Pract. 2020 May 21;7(5):531-542. doi: 10.1002/mdc3.12968. - Titova N, Chaudhuri KR. Palliative Care and Nonmotor Symptoms in Parkinson's Disease and Parkinsonism. Int Rev Neurobiol. 2017;134:1239-1255. doi: 10.1016/bs.irn.2017.05.014. - Birnbacher, D. (2014). Sterbefasten – eine ethische Bewertung. Humanistischer Pressedienst. - Burgos, R., Bretón, I., Cereda, E., Desport, J., Dziewas, R., L., G., et al. (2018). ESPEN guideline clinical nutrition in neurology. Clinical Nutrition. - Elena Klinik Kassel, S. (kein Datum). Schluckstörung bei Parkinson; Invormationsblatt für Patienten und Angehörige. - Evans, S., Soar, N., Lang, A., P., S., Archer, S., & Birns, J. (28. November 2019). Risk feeding in the advanced stages of Parkinson’s disease. Progress in Neurology and Psychiatry. - Goldman, J., & Postuma, R. (August 2014). Premotor and non-motor features of Parkinson`s disease. Current Opinion in Neurology, S. 434-4441. - Höglinger G. U. (2018). Parkinson-Syndrome kompakt. Thieme. - Jox, R., Black, I., Borasio, G. D., & Anneser, J. (2017). Voluntary stopping of eating and drinking: is medical support ethically justified? BMC Medicin. - Manor, Y., Giladi, N., Cohen, A., Fliss, D., & Cohen, J. (15. October 2007). Validation of a swallowing disturbance questionnaire for detecting dysphagia in patients with Parkinson's disease. Movement disorders, S. 1917-21. - Myrte E. Hamburg, C. F. (31. Januar 2014). Food for love: the role of food offering in empathic emotion regulation. Frontiers in Psychology. - Parkinson`s, U. (March 2018). Diet and Parkinson`s. Brochure. |
| **Assessment** | - Topic related Discussion/Exercise - Self-Assessment (20 multiple choice questions) |

| **Lesson Plan** | |
| --- | --- |
| **Teaching Unit** | Loss, Grief management and Bereavement |
| **Title** | **Week 10: Loss, Grief management and Bereavement** |
| **Learning Objectives** | Identify and describe the losses (and their impact) that patients and their families face across the illness trajectory and (for families) after death.  Differentiate between loss, grief, bereavement and mourning, including triggers for abnormal of prolonged grief reactions.  Identify common loss/grief models and describe their value for practice, including identification of complex grief. |
| **Summary &**  **scheduling** | Focuses on loss, grief and bereavement. It includes the definitions, explains the healthcare relevant management strategies, and inspects helpful and unhelpful coping strategies. |
| **Learning Content A - Attitude K - Knowledge S - Skills** | Attitude: Participants understand how people deal with the loss. They also appreciate that grief is a natural process which should not be medicalised.  Knowledge: Participants will get to know about:   - Definitions of loss, grief, mourning, bereavement. - Theories and types of loss, grief and bereavement. - The interplay of loss and grief. - The different aspects and stages of grief. - Coping strategies. - Clinical indications of poor coping.   Skills: Healthcare professionals will be able to identify grief patterns. They will also be able to use the clinical indicators of poor coping and intervene as necessary. |
| **Teaching Methods** | - Presentation (slides) - Video component - Reading materials |
| **Literature** | - Gofton TE, Chum M, Schulz V, et al. Challenges facing palliative neurology practice: A qualitative analysis. Journal of the Neurological Sciences 2018;385:225-31. - Fox S, Azman A, Timmons S. Palliative care needs in Parkinson’s disease: focus on anticipatory grief in family carers. Ann Palliat Med 2020;9(Suppl 1):S34-S43. - Ryan K, Connolly M, Charnley K, Ainscough A, Crinion J, Hayden C, Keegan O, Larkin P, Lynch M, McEvoy D, McQuillan R, O’Donoghue L, O’Hanlon M, Reaper-Reynolds S, Regan J, Rowe D, Wynne M; Palliative Care Competence Framework Steering Group. (2014). *Palliative Care Competence Framework*. Dublin: Health Service Executive - Ma HI, Saint-Hilaire M, Thomas CA, Tickle-Degnen L. Stigma as a key determinant of health-related quality of life in Parkinson’s disease. Qual Life Res. 2016. 25(12):3037-3045. - Oehlberg K, Barg FK, Brown GK, Taraborelli D, Stern MB, Weintraub D. Attitudes regarding the etiology and treatment of depression in Parkinson’s disease: a qualitative study. J Geriatr Psychiatry Neurol. 2008. 21(2):123-32. - Penner LA.& Roger K 2012. The person in the room: how relating holistically contributes to an effective patient-care provider alliance. *Communication & Medicine,* 9**,** 49-58. - Diane Wepa (Ed.), *Cultural Safety in Aotearoa New Zealand* (2^nd^ ed). 2015. - Bowlby, J. (1980) *Attachment and Loss Vol. 3* London: Pelican Books - Doka KJ (1989) *Disenfranchised Grief: Recognizing Hidden Sorrow*. Lexington: Lexington Books - Silverman and Klass (1996) Continuing bonds. New Understandings of grief. Phil.PA, USA: Taylor and Francis. - Kübler-Ross E (1969) On Death and Dying, Macmillan, New York NY - Kübler-Ross, E. and Kessler, D. (2005) *On Grief and Grieving*. London: Bath Press/Simon & Schuster - Rando, T.A (1993) Treatment of Complicated Mourning IL: Research Press. - Stroebe, M.& Schut, H. (1999) The dual process model of coping with bereavement: rationale and description. Death Studies, Vol.23 pp 197-224 - Worden, J.W. (2003) Grief Counselling and Grief Therapy. 3rd ed. Hove: Brunner-Routledge |
| **Assessment** | - Topic related Discussion/Exercise - Self-Assessment (20 multiple choice questions) |

| **Lesson Plan** | |
| --- | --- |
| **Teaching Unit** | Spiritual Care |
| **Title** | **Week 11: Spiritual Care** |
| **Learning Objectives** | Understand the concepts of spirituality, spiritual needs and care.  Be able to apply the spiritual care model throughout the illness course. |
| **Summary &**  **scheduling** | It introduces basic concepts, such as spiritual dimension, spirituality and presents a comprehensive spiritual care model for managing PD. |
| **Learning Content A - Attitude K - Knowledge S - Skills** | Attitude: Participants will also tap into the spiritual dimension sensibly so as not to cause false hopes or increasing vulnerability in patients with PD and their caregivers.  Knowledge:   - The terms spiritual dimension, spirituality, and spiritual care - The impact of spirituality and unmet spiritual needs to patients‘ and their caregivers lives - The meaning of patient-caregiver centred care model(s) - The importance of self-care strategies and support for professionals   Skills: Healthcare professionals will be prompted to explore their own spirituality also as a defence to emotional cookout/burnout whist providing palliative and terminal care. |
| **Teaching Methods** | - Presentation (slides) - Video component - Reading materials |
| **Literature** | - Best M. Dignity in Palliative Care. In: MacLeod RD, Van den Block L. editors. Textbook of Palliative Care. Springer Nature Switzerland AG, 2019:1-11. - Best M, Leget C, Goodhead A, et al. An EAPC white paper on multi-disciplinary education for spiritual care in palliative care. BMC Palliat Care 2020;19:9. - Centeno C, Arias-Casais N. Global palliative care: from need to action. Lancet Glob Health 2019;7:e815-6. - CHANG, GITLIN D., PATEL R., 2011, The depressed patient and suicidal patient in the emergency department: evidence-based management and treatment strategies, in Emergency medicine practice, vol. 13, nº 9 - Chirico F. Spiritual well-being in the 21st century: It is time to review the current WHO’s health definition. J Health Soc Sci 2016;1:11-6. - Chochinov HM, Hack T, Hassard T, et al. Dignity therapy: a novel psychotherapeutic intervention for patients near the end of life. J Clin Oncol 2005;23:5520-5. - Gamondi C, Larkin P, Payne S. Core competencies in palliative care: an EAPC white paper on palliative care education: part 2. Eur J Palliat Care. 2013. - Harris DA, Jack K, Wibberley C. The meaning of living with uncertainty for people with motor neurone disease. J Clin Nurs 2018;27:2062-71. - Kleinman A. Patients and Healers in the Context of Culture. An Exploration of the Borderland between Anthropology, Medicine, and Psychiatry. Berkeley, Los Angeles & London: University of California Press; 1980. - Kleinman A. The Illness Narrative. Suffering, Healing & the Human Condition. New York: Basic Books; 1988. - Lipscomb J, Gotay C, Snyder C. editors. Outcomes Assessment in Cancer: Measures, Methods and Applications. Cambridge: Cambridge University Press; 2004. - McSherry W, Smith J. Spiritual Care. In: McSherry W, McSherry R, Watson R, editors. Care in Nursing: Principles, Values and Skills. Oxford Oxford University Press; 2012. p. 118. - Paal P, Lex KM, Brandstötter C, Weck C, Lorenzl S. Spiritual care as an integrated approach to palliative care for patients with neurodegenerative diseases and their caregivers: a literature review. Ann Palliat Med 2020. - Penner LA, Roger K. The person in the room: how relating holistically contributes to an effective patient-care provider alliance. Commun Med 2012;9:49-58. - Piderman KM, Radecki Breitkopf C, Jenkins SM, et al. The impact of a spiritual legacy intervention in patients with brain cancers and other neurologic illnesses and their support persons. Psychooncology 2017;26:346-53. - Roger K, Wetzel M, Hutchinson S, et al. "How can I still be me?": Strategies to maintain a sense of self in the context of a neurological condition. Int J Qual Stud Health Well-being 2014;9:23534. - Sharpe M, Stone J, Hibberd C, et al. Neurology out- patients with symptoms unexplained by disease: illness beliefs and financial benefits predict 1-year outcome. Psychological Medicine 2010;40:689-98. - Snyder J, Adams K, Crooks VA, et al. "I knew what was going to happen if I did nothing and so I was going to  do something": faith, hope, and trust in the decisions  of Canadians with multiple sclerosis to seek unproven interventions abroad. BMC Health Serv Res 2014;14:445. |
| **Assessment** | - Topic related Discussion/Exercise - Self-Assessment (20 multiple choice questions) |

| **Lesson Plan** | |
| --- | --- |
| **Teaching Unit** | Testimonials and Discussion on Advanced Directives |
| **Title** | **Week 12: Testimonials and Discussion on Advanced Directives** |
| **Learning Objectives** | Comprehend patients’ and caregivers’ views on advance care planning.  Understand different perspectives in complex, advance care decisions. |
| **Summary &**  **scheduling** | Stories from patients and families describing how they reached their ACP decisions, how satisfied they are with these decisions, and why they feel that way (3 hours). |
| **Learning Content A - Attitude K - Knowledge S - Skills** | Attitude:  Participants are encouraged to collaborate to identify values, goals, and preferences early, as well as throughout the disease trajectory, to facilitate care concordant with patients’ preferences to maintain their quality of life.  Knowledge: Participants will have improved knowledge of how:   - PD-related life changes impact daily life activities and affect future planning, including advance care - Support and advice should be provided for life changes within a structured decision-making process - Important the personalization of the provided information and support (no ‘one-size-fits-all’ models) is for patients and their caregivers   Skills: Enable patients and their caregivers to make informed decisions in collaboration with their formal healthcare providers. |
| **Teaching Methods** | - Presentation (slides) - Video component - Reading materials |
| **Literature** | - Lum HD, Jordan SR, Brungardt A, Ayele R, Katz M, Miyasaki JM, Hall A, Jones J, Kluger B. Framing advance care planning in Parkinson disease: Patient and care partner perspectives. Neurology. 2019 May 28;92(22):e2571-e2579. doi: 10.1212/WNL.0000000000007552. Epub 2019 Apr 26. PMID: 31028124; PMCID: PMC6556088. - Armstrong MJ, Alliance S, Taylor A, Corsentino P, Galvin JE. End-of-life experiences in dementia with Lewy bodies: Qualitative interviews with former caregivers. PLoS One. 2019 May 30;14(5):e0217039. doi: 10.1371/journal.pone.0217039. PMID: 31145749; PMCID: PMC6542529. - Sokol LL, Young MJ, Paparian J, Kluger BM, Lum HD, Besbris J, Kramer NM, Lang AE, Espay AJ, Dubaz OM, Miyasaki JM, Matlock DD, Simuni T, Cerf M. Advance care planning in Parkinson's disease: ethical challenges and future directions. NPJ Parkinsons Dis. 2019 Nov 22;5:24. doi: 10.1038/s41531-019-0098-0. PMID: 31799376; PMCID: PMC6874532. - Tuck KK, Brod L, Nutt J, Fromme EK. Preferences of patients with Parkinson's disease for communication about advanced care planning. Am J Hosp Palliat Care. 2015 Feb;32(1):68-77. doi: 10.1177/1049909113504241. Epub 2013 Sep 19. PMID: 24052430. |
| **Assessment** | - Topic related Discussion/Exercise - Self-Assessment (20 multiple choice questions) |
